# Heterogeneous loss of HIV transcription and proviral DNA from 8E5/LAV lymphoblastic leukemia cells revealed by RNA FISH:FLOW analyses

**DOI:** 10.1186/s12977-016-0289-2

**Published:** 2016-08-11

**Authors:** Kaley M. Wilburn, Henry C. Mwandumba, Kondwani C. Jambo, Saikat Boliar, Sabrina Solouki, David G. Russell, David W. Gludish

**Affiliations:** 1 Department of Microbiology and Immunology, College of Veterinary Medicine, Cornell University, Ithaca, NY 14853 USA; 2 Malawi-Liverpool-Wellcome Trust Clinical Research Programme, University of Malawi College of Medicine, Blantyre, Malawi; 3 Department of Clinical Sciences, Liverpool School of Tropical Medicine, Liverpool, UK

**Keywords:** 8E5, Reservoir quantitation, FISH:FLOW, FISH:FACS, HIV cell-associated RNA, HIV flow cytometry assay, HIV proviral silencing, HIV proviral loss, HIV RNA in situ hybridization

## Abstract

8E5/LAV cells harbor a single HIV provirus, and are used frequently to generate standards for HIV genome quantification. Using flow cytometry-based in situ mRNA hybridization validated by qPCR, we find that different batches of 8E5 cells contain varying numbers of cells lacking viral mRNA and/or viral genomes. These findings raise concerns for studies employing 8E5 cells for quantitation, and highlight the value of mRNA FISH and flow cytometry in the detection and enumeration of HIV-positive cells.

## Main text

A significant challenge in the search for a cure for HIV is the quantification of latently infected reservoir cells. The rarity of these cells in the blood of ART-suppressed patients renders assessment of the size of the latent reservoir extremely challenging [1]. Current methods to estimate the number of cell-associated viral genomes usually involve PCR-based amplification of integrated proviral DNA or viral RNA and their comparison with cell-based standards. The human lymphoblastic leukemia cell clone 8E5/LAV (8E5) is widely employed for such studies [2, 3], and harbors a single integrated HIV provirus that drives production of defective virions [3, 4]. These cells are routinely mixed with HIV-uninfected lymphoblasts in known proportions to generate standard curves, to facilitate relative quantification of viral genomes using the number of cycles of PCR amplification.

**Fig. 1:**
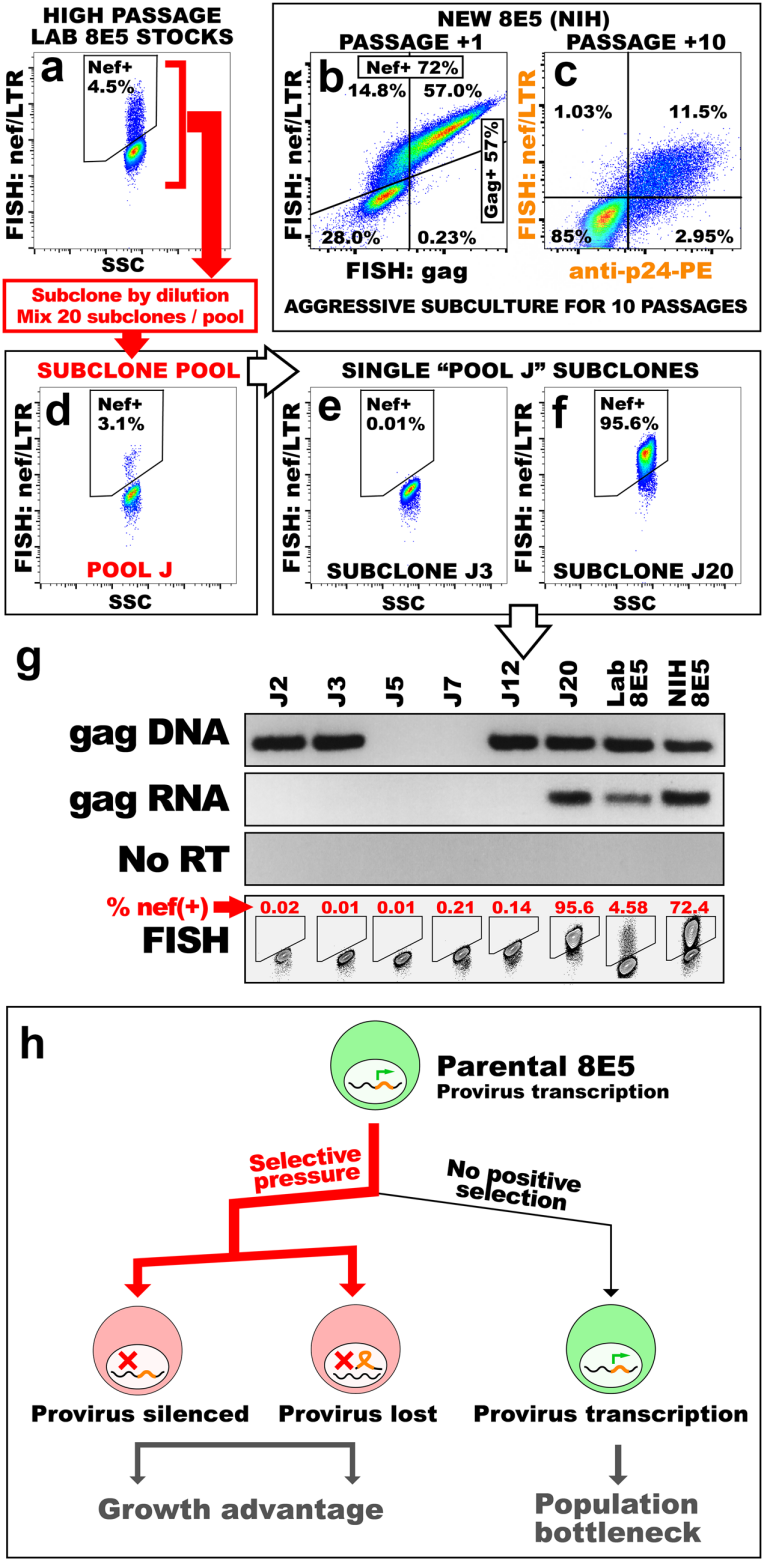
Variable infection of HIV in 8E5 cells. **a**–**c** FISH probes detect *nef/LTR* RNA in the indicated subpopulations (Nef+) of 8E5 cells either from archival laboratory stocks (**a**, originally purchased from ATCC) or newly acquired **(b, c)** through the NIH AIDS Reagent Program. FISH:FLOW *dot plots* show that HIV *nef/LTR* probe signal correlates in the same cells with HIV gag mRNA **(b)** and p24 antibody staining **(c)**. Cells in **(c)** were obtained by repeated high-ratio subculture of the new cell stock from **(b)**. **d** 8E5 subclones were generated and combined into pools of 20; these pools were screened for HIV-transcribing subclones; Pool J is shown, harboring likely a single clone of 100 % HIV penetrance (<5 % of total cells in the pool are HIV *nef/LTR* positive). **e**–**g** Analyses of HIV mRNA and proviral DNA in single J-pool subclones. Clone J3 (**e**) harbors no *nef/LTR* transcript detected by RNA FISH, while J20 (**f**) is uniformly *nef/LTR*-positive. **g** (*Top 3 panels*) Proviral gag DNA qPCR and corresponding gag mRNA qRT-PCR with no reverse transcriptase cDNA controls. **g** (*Bottom panel*) FISH:FLOW contour plots for *nef/LTR* RNA from selected J-pool subclones. Frequencies of *nef/LTR*-positive cells indicated in red. **h** Model for 8E5 cell population dynamics. Parental 8E5 cells are under strong selective pressure to exclude or silence the HIV provirus. Cells that achieve these outcomes (*red*) acquire significant growth or survival advantages. During long-term culture, this advantage will shift the 8E5 population, leading to a bottleneck of HIV-expressing cells (*green*) in the culture

We recently developed a method to detect HIV RNA-positive cells in patient samples by fluorescence in situ hybridization and flow cytometry (FISH:FLOW) [5]. Short (25nt), tagged ssDNA oligo probes were generated to cover ~600–1400 bp of the HIV *gag* or *nef* open reading frames from ADA (*nef/LTR*, 8794-9407; *gag*, 823-2240 LGC Biosearch). These probes are hybridized to RNA in PFA-fixed and ethanol-permeabilized cells providing HIV RNA readout with cellular resolution via flow cytometry. To test the limits of this assay, we generated a dilution series of 8E5/CEM cell standards and analyzed them by FISH:FLOW. Surprisingly, we found the 8E5 population was heterogeneous with only 4.5 % of cells positive for HIV *nef/LTR* and 2.9 % for *gag* transcripts detected by RNA FISH (Fig. 1a and data not shown). Dilutions of the 8E5 cells yielded linear standard plots (not shown), as might also be expected from PCR analysis of comparable dilution series. However, the absolute number of viral genomes inferred by this method would underestimate the true values obtained using standards that assumed 100 % of 8E5 cells in the starting population were HIV-infected.

HIV transcription may not proceed in all 8E5 cells at all points in the cell cycle, a scenario that could explain a subpopulation of HIV RNA-negative cells in 8E5 cultures. However, given the observed low fraction of positive cells, we reasoned that proviral loss or durable silencing [6, 7] would more likely account for a majority of cells negative for HIV *nef/LTR* or *gag* mRNA. To assess the maintenance of the HIV proviral genome in the 8E5 cells we generated 200 subclones by limiting dilution, and expanded these cells for analyses by RNA FISH. We first combined the subclones into pools of 20, and screened for HIV positive pools. To our surprise, only one subclone pool (Pool J) showed signal for HIV *nef/LTR* at a frequency suggesting that only a single clone in the pool was positive (Fig. 1d). We then analyzed subclones individually and found that most clones were entirely negative for *nef/LTR* RNA (representative clone J3, Fig. 1e). By contrast, clone J20 was homogeneously positive for HIV *nef/LTR* RNA by FISH:FLOW analyses (Fig. 1f). The segregation of positive and negative clones is clearly visible in the FISH contour plots (Fig. 1g, bottom panel). FISH:FLOW analysis with HIV *gag* RNA probes yielded similar results (data not shown). Interestingly, the lower than expected frequency of HIV-transcribing clones (1/200 vs. ~4.5/100 expected) suggests that cells containing active HIV proviral genomes are at a survival or clonogenic disadvantage compared to those that have silenced or lost the provirus.

Absence of HIV transcription could result from proviral silencing or loss of proviral genomic DNA. Either scenario could be the product of negative selective pressure experienced by HIV-infected lymphoblasts in long-term culture. Transcriptionally silent or HIV-negative subclones within the 8E5 population would have a growth advantage, and would rapidly outcompete the HIV-expressing population. To address this experimentally we compared relative frequencies of proviral DNA (Qiagen QIamp) and HIV *gag* mRNA (Trizol) by qPCR and qRT-PCR (BioRad iTAQ). Two independent regions of *gag* were amplified and normalized to GAPDH genomic DNA or cDNA from the same sample (Fig. 1g, GAPDH data not shown). Intriguingly, some subclones lacking HIV *nef/LTR* transcripts still harbored gag proviral DNA, while in other subclones the HIV provirus was undetectable. Possible genomic DNA contamination was ruled out using controls lacking reverse transcriptase (no RT, Fig. 1g). These data indicate that both proviral genome silencing and genome deletion are occurring in 8E5 cells maintained in culture. Interestingly, the LAV provirus in 8E5 is integrated at chromosome 13q14-q21 [8], a site containing common fragile sites that would render this clone susceptible to proviral loss by genomic instability.

We acquired a fresh aliquot of 8E5 cells from the HIV AIDS Reagent Program to determine whether population heterogeneity might be a consistent feature of these cells. These 8E5 cells were tested by *nef/LTR* and *gag* RNA FISH within 5 days of their establishment in culture. The *nef*-positive gate constituted the main population of cells (72 %, Fig. 1b). Importantly, no cells stained positive for *gag* RNA without also expressing *nef/LTR* RNA in this multiplex assay (Fig. 1b) as would be expected based on the staged transcription of HIV. This representation highlights the ability of FISH:FLOW to discern among different stages of HIV infection (*gag* heterogeneity in the *nef* + population). Subsequent passaging of the newly obtained cells at a 1:10 ratio led, by passage 10, to the reduction of *nef/LTR* and *gag* RNA-positive cells below 50 % (not shown). Aggressive subculture by splitting the cells very low (bottleneck founder effect) accelerated the loss of *nef/LTR* transcription, where only 15 % of 8E5 cells fresh from a public repository transcribed HIV *nef/LTR* by passage 10 (Fig. 1c); importantly almost all of the *nef*-positive cells also stained p24-positive (Beckman anti-p24 KC57) (Fig. 1c). These data demonstrate FISH:FLOW is a surrogate assay for standard measures of HIV production and that the HIV transcriptional loss documented in 8E5 subclones is a reproducible characteristic of this cell line. We believe this observation reflects the strong selection of founder subclones that achieve spontaneous loss of HIV proviral DNA.

Together our data support a model (Fig. 1h) where 8E5 cells acquire a selective advantage in continuous cell culture if they extinguish HIV expression, either by transcriptional silencing or by proviral genome loss. We suspect that stressing the cells during culture through delayed passage is likely to exacerbate this behavior, hastening a bottleneck of HIV-positive cells in the population. The loss of HIV from 8E5 cultures is of practical significance considering the widespread utilization of these cells for quantitation of HIV genome abundance in patient samples. Our findings appear consistent with cautions raised in a recent analysis, which found variable numbers of proviral insertions within common latently infected cell lines previously assumed to be homogeneous [9]. Our findings highlight the robust data these cell lines yield and underscore their intrinsic value to the field, but also support recent initiatives to validate common reagents in pursuit of reproducibility. The FISH:FLOW method we present here is a convenient means to rigorously validate the penetrance of HIV in the 8E5 starting population in any laboratory with access to a flow cytometer; without such validation, we suggest that quantification of HIV genomes using 8E5 cells should be restricted to relative comparisons only.

Worldwide, investigators studying persistence and therapeutic reactivation of latent HIV reservoirs are heavily invested in PCR readouts of genome quantitation. The FISH:FLOW method we have developed is one example of the very few tools that allow quantification of HIV infection at the level of the individual cell; importantly this resolution is lost in PCR studies of cell-associated DNA or RNA from bulk populations. We feel strongly that a critical mass of laboratories investing in cell-level tools has not yet been reached, and the simple results presented here using a common cell line highlight the value of such studies as a complement to PCR approaches. Importantly, combining FISH:FLOW with antibody surface phenotyping meets the evermore urgent need to characterize specific cell subsets that harbor HIV in vivo. Multiple probe colors can assay different HIV transcripts in a single cell, allowing one to discern between early and late stage transcription, and increases the confidence that positive signal corresponds to intact provirus. The ability to then FACS-purify infected cells from human patient samples and study their transcriptional profile adds functional genomics to the growing list of possibilities. We feel these are opportunities not to be missed in context of the current challenges facing the field of HIV persistence and eradication.
